# *larvalign*: Aligning Gene Expression Patterns from the Larval Brain of *Drosophila melanogaster*

**DOI:** 10.1007/s12021-017-9349-6

**Published:** 2017-11-10

**Authors:** Sascha E. A. Muenzing, Martin Strauch, James W. Truman, Katja Bühler, Andreas S. Thum, Dorit Merhof

**Affiliations:** 1 Institute of Imaging & Computer Vision, RWTH Aachen University, Aachen, Germany; 2 Present Address: Forschungszentrum Jülich, Institute of Neuroscience and Medicine, Jülich, Germany; 3 Janelia Research Campus, Howard Hughes Medical Institute, Ashburn, VA USA; 4 Friday Harbor Laboratories, University of Washington, Friday Harbor, WA USA; 5 VRVis Zentrum für Virtual Reality und Visualisierung Forschungs-GmbH, Vienna, Austria; 6 Department of Biology, University of Konstanz, Constance, Germany; 7 Zukunftskolleg, University of Konstanz, Constance, Germany; 8 Present Address: Department of Genetics, University of Leipzig, Leipzig, Germany

**Keywords:** *Drosophila melanogaster*, Larval brain, Standard brain, Image registration, *elastix*, Gene expression patterns

## Abstract

The larval brain of the fruit fly *Drosophila melanogaster* is a small, tractable model system for neuroscience. Genes for fluorescent marker proteins can be expressed in defined, spatially restricted neuron populations. Here, we introduce the methods for 1) generating a standard template of the larval central nervous system (CNS), 2) spatial mapping of expression patterns from different larvae into a reference space defined by the standard template. We provide a manually annotated gold standard that serves for evaluation of the registration framework involved in template generation and mapping. A method for registration quality assessment enables the automatic detection of registration errors, and a semi-automatic registration method allows one to correct registrations, which is a prerequisite for a high-quality, curated database of expression patterns. All computational methods are available within the *larvalign* software package: https://github.com/larvalign/larvalign/releases/tag/v1.0

## Introduction

Due to the availability of an extensive genetic toolkit, the fruit fly *Drosophila melanogaster* has become one of the preferred model organisms in neuroscience. The brain of the fruit fly larva consists of only about 10,000 (Nassif et al. 2003) neurons, which renders it a tractable model system that, despite its simplicity, shares many of the structural features of the adult. In combination with the considerable behavioural repertoire that even the fruit fly larva possesses, this allows studies on behavioural responses to sensory stimuli, such as odors (Gerber and Stocker 2007) and light (Keene and Sprecher 2012).

Genetic tools are widely used also in the study of the larval brain. For example, the GAL4/UAS system serves to express a marker, such as the green fluorescent protein (GFP), in a defined subpopulation of the neurons (Brand and Perrimon 1993; Venken et al. 2011).

Here, we develop the computational methods for displaying such gene expression patterns from different larval brains in a common reference space. Based on microscope images of brain anatomy*, neuropil* (NP, anti-N-cadherin antibody*)* staining and *nerve tract* (NT, anti-neuroglian antibody) staining, we construct a standard brain *template* onto which the anatomical NP channels from different individuals are registered. Using the transformations thus obtained, the NT and the *gene expression* (GE) channels from these individuals can subsequently be mapped into a common reference space.

Both the generation of the brain template and the mapping of individuals onto the template rely on image registration. In this work, we propose and evaluate computational methods that are robust in the face of biological and experimental variation, such as brain deformations, inhomogeneous staining, and inconsistencies in sample preparation for data recorded over a long period of time.

Image registration is a highly data dependent process. However, there is no generic image registration approach that works best for all applications, that is, for each application a specialized registration algorithm or registration framework has to be developed. We opted to develop a registration framework based upon a well-established generic state-of-the-art registration algorithm. The core of an image registration framework lies in the actual registration algorithm, which typically consists of different components such as transformation models, similarity measures, and regularization and optimization techniques. Optimal combination and parametrization of these components is crucial for obtaining the best possible image registration accuracy (Sotiras et al. 2013; Viergever et al. 2016; Tustison et al. 2013).

The software package *larvalign* presents a computational framework, that is, a dedicated sequence of image processing methods optimized for the registration of microscope images of the CNS of the third instar Drosophila larva. *larvalign* utilizes open source software toolkits for image processing (*elastix*, ITK-SNAP, Fiji). The image processing pipelines for image registration and quality assessment are implemented and bundled in Matlab, providing an installable self-contained software whose functionalities can be accessed programmatically or by a graphical user interface for easy and convenient conduction of single scan and batch registration.

In the following, we introduce a gold standard for evaluation and then describe our registration framework (Materials and Methods), followed by extensive empirical evaluation and biological use cases (Results) demonstrating the practical applicability of the method for the mapping of gene expression patterns.

## Material and Methods

### Gold Standard for Evaluation of Registration Accuracy

Image registration is the spatial mapping of each voxel in one image onto the corresponding voxel in the other image. In order to evaluate the complete spatial mapping obtained by image registration, the true underlying spatial correspondence of each voxel between two images has to be known, the *ground truth* (Pluim et al. 2016). In biomedical image registration applications, such a ground truth is usually not available. However, it is possible to establish point-wise or regional correspondences between images by assigning landmarks at distinct positions of the imaged object or by segmenting distinct regions, typically related to the anatomical structure of the imaged object. In image registration, such a set of spatial correspondences is called a *gold standard*. Having a gold standard available, one can assess the registration accuracy by calculation of the Euclidean distance between the position of the reference landmark and the position computed by the registration algorithm. In the following we refer to this measure for registration accuracy by the term Landmark Registration Error (LRE). In contrast to a ground truth, a gold standard inevitably contains a certain annotation error due to biological variation and image quality (limited spatial image resolution, image acquisition artifacts etc.) and is affected by inter-, and intra-rater deviation in case of manual annotation.

**Fig. 1:**
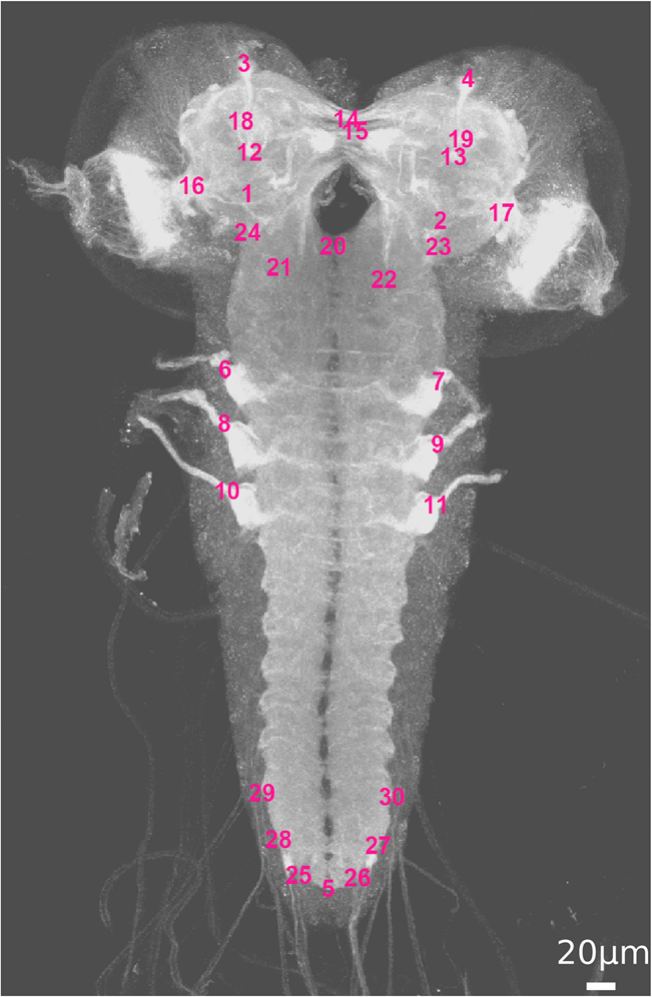
Maximum intensity projection (MIP) of an exemplary scan with annotated landmarks. For landmark labels, see Table 1

An expert in neurobiology (co-author AT) selected landmark positions that are expected to be clearly visible in most scans, to have little anatomical variance, and to be located at relevant regions of neural pathways. In this way we defined 30 anatomical landmark positions covering the entire larval CNS: Fig. 1. The diameter of the anatomical structures at the selected landmarks – often nerve entry points – is in the range of 2-8 μm, with more than 50% of the structures having diameters >5 μm.

In order to assess the intra-rater variability of the landmark annotation, four scans were annotated twice by the same neurobiologist (AT, co-author). For these four exemplary scans, the mean intra-rater deviation over all landmarks per scan is 2.0 ± 1.2 μm and the maximum deviation is 3.5 ± 1.8 μm. Table 1 lists the intra-rater deviation per landmark and per scan.

**Table 1:** Intra-rater variance. For each of four scans, all 30 landmarks (landmark positions: see Fig. 1) were annotated twice by the same expert. The table shows the deviation (in μm) between the two annotations for each landmark/scan combination. Additionally, we report mean ± standard deviation and the maximum for each landmark

***#***	Landmark label	Scan 1	Scan 2	Scan 3	Scan 4	Mean ± Std		Max
1	left antennal nerve	3.0	3.0	0.5	0.7	1.8	1.4	3.0
2	right antennal nerve	2.6	3.4	0.5	2.9	2.3	1.3	3.4
3	left tip of vertical lobe	2.6	2.2	1.8	3.8	2.6	0.9	3.8
4	right tip of vertical lobe	2.2	0.5	1.5	0.5	1.2	0.9	2.2
5	end of ventral nerve cord	0.5	4.1	2.1	0.7	1.8	1.7	4.1
6	left thoracic nerve entry T1	2.1	0.6	0.9	2.0	1.4	0.7	2.1
7	right thoracic nerve entry T1	1.3	0.5	1.3	1.0	1.0	0.4	1.3
8	left thoracic nerve entry T2	1.0	0.5	1.3	0.7	0.9	0.4	1.3
9	right thoracic nerve entry T2	3.4	3.9	2.4	1.5	2.8	1.1	3.9
10	left thoracic nerve entry T3	0.6	0.6	1.0	0.9	0.8	0.2	1.0
11	right thoracic nerve entry T3	5.6	2.1	1.0	2.3	2.7	2.0	5.6
12	left upper peduncle	3.1	3.3	2.9	6.2	3.9	1.6	6.2
13	right upper peduncle	5.6	4.4	2.1	3.6	3.9	1.5	5.6
14	anterior upper commisure	4.2	1.0	1.5	2.9	2.4	1.5	4.2
15	posterior upper commisure	0.0	0.6	3.9	2.3	1.7	1.8	3.9
16	left anterior LON nerve	1.0	2.5	2.0	2.3	1.9	0.6	2.5
17	right anterior LON nerve	0.6	1.4	4.4	2.3	2.2	1.6	4.4
18	left MB vertical medial lobe connection	0.5	0.5	0.5	2.9	1.1	1.2	2.9
19	right MB vertical medial lobe connection	2.4	1.4	2.7	0.5	1.7	1.0	2.7
20	center SEZ neuropil fusion	1.6	4.3	1.4	1.8	2.3	1.3	4.3
21	left upper most anterior nerve entry	0.9	2.2	2.4	4.3	2.5	1.4	4.3
22	right upper most anterior nerve entry	10.0	1.0	2.7	5.4	4.8	3.9	*10.0 (overall max)*
23	right basal brain neuropil border posterior	0.5	2.2	1.4	2.1	1.5	0.8	2.2
24	left basal brain neuropil border posterior	4.2	0.6	4.2	2.3	2.8	1.7	4.2
25	left A8 nerve entry	0.0	1.8	1.5	4.4	1.9	1.8	4.4
26	right A8 nerve entry	1.8	1.4	1.0	4.6	2.2	1.6	4.6
27	right A7 nerve entry	0.0	0.6	1.3	1.0	0.7	0.6	1.3
28	left A7 nerve entry	1.0	0.5	0.5	1.4	0.8	0.5	1.4
29	left A6 nerve entry	2.1	0.5	0.5	0.7	0.9	0.8	2.1
30	right A6 nerve entry	0.5	2.1	0.0	2.8	1.3	1.3	2.8
	Average	2.2	1.8	1.7	2.4	2.0	1.2	3.5 ± 1.8

#### Additional Visual Quality Assessment

Additional quality assessment by an expert (co-author AT) involved a visual inspection process guided by the following 6 error categories. Only registrations without any error of the type 1–6 were accepted.

1. The thoracic segments in the VNC were shifted.
2. The ventral nerve cord (VNC) was stretched beyond the standard brain border.
3. A brain hemisphere at the level of the developing optic lobes was misaligned.
4. The entire alignment of subject and target was wrong, i.e. both brain hemispheres were extremely stretched and multiple misalignments were present in the VNC.
5. The VNC was shifted to the left or right side.
6. There was no neuropil staining and thus no possibility for the program to register the brain scan.

### Registration Algorithms

Typically, image registration is performed in two stages: the global alignment of images (linear transformation), and the local alignment (non-linear transformation), the so-called deformable image registration. For the deformable registration one can distinguish between non-parametric methods (variational methods) and parametric methods (mostly B-Spline models). For both categories, we investigated the respective most promising state-of-the-art toolkit for biomedical image registration, *ANTs* (Avants et al. 2011) and *elastix* (Klein et al. 2010). Both have been employed successfully for image registration of human, rat, and mouse brains (Klein et al. 2009a; Murphy et al. 2011).

ANTs is a state-of-the-art medical image registration and segmentation toolkit. It contains algorithms for image registration with various transformation models (rigid, affine, elastic, diffeomorphic, symmetric) and similarity metrics (cross-correlation, mutual information, landmarks etc.). ANTs image registration is probably best known for its symmetric diffeomorphic registration algorithm (SyN) (Avants et al. 2008). In an evaluation study of 14 nonlinear deformation algorithms applied to human brain MRI registration, SyN was one of two algorithms that achieved the best registration accuracy (Klein et al. 2009a). However, it should be noted that the *elastix* toolkit was not yet available at the time of this evaluation study.

The *elastix* image registration toolkit consists of a collection of state-of-the-art algorithms that are commonly used to solve (medical) image registration problems (Klein et al. 2010; Shamonin et al. 2014). Similar to ANTs, it provides various transformation models, similarity measures, optimization methods as well as a GPU implementation.

ANTs and *elastix* are open source software and are based on the Insight Segmentation and Registration Toolkit (ITK, https://itk.org/), a widely used medical image processing library of the National Library of Medicine.

We conducted an initial registration experiment to compare the ANTs SyN and the *elastix* B-Spline registration approach for the pairwise (subject-to-subject) registration on our image data. We registered each of the initially annotated four scans to each other, yielding six combinations of pairwise registrations. The registration setup used, is described in the following.

#### Global (Linear) Registration

The global registration (linear transformation) serves as an initialization for the deformable registration (non-linear transformation) step. The aim of the global registration is to match the orientation and size of the larval brains in both images. Both, the ANTs and the *elastix* toolbox, provide an affine transformation method and a normalized cross correlation (NCC) or mutual information (MI) similarity metric.

In general, brain samples may be positioned arbitrarily on the object plate and thus the images may display the brain sample with large rotations and/or in flipped Z-direction. The images used here did not show large rotations and were inverted, if necessary, in a preprocessing step. We found that an affine transformation aligned the images well regarding orientation and size. The NCC similarity measure performed better than the MI similarity measure with respect to LRE. We therefore used an affine transformation and the NCC similarity measure for the global registration with ANTs and *elastix*.

#### Local (Non-Linear) Registration with ANTs

We tried to optimize the setup of the nonlinear registration in order to find the best (smallest LRE) configuration for both the ANTs and the *elastix* approach for confocal images of the larval *Drosophila* brain. A multi-resolution scheme of five levels and the NCC similarity measure was used for both approaches.

We employed the SyN registration method of the ANTs toolbox (Avants et al. 2011). We evaluated the registration accuracy for varying values of the parameter defining the regularization of the velocity field. The best registration accuracy in terms of the landmark registration error, was achieved with values between 24 and 28 for the regularization parameter <r > (cf. parameter settings listed below) Using the default parameter value of 3, one registration takes about 8 h (Intel(R) Core(TM) i7-6700 K CPU @ 4.00GHz, 64GB RAM), and with larger values of the parameter <r > the computation increases further.

antsRegistration, release 2.1:

- initial-moving-transform [<referenceImage>,<subjectImage>, 1]
- transform Affine[0.1]
- metric GC[<referenceImage>,<subjectImage>, 1, 0, Regular, 0.25]
- convergence [500x500x500x500, 1e-6,10] --smoothing-sigmas 4x2x1x0 --shrink-factors 8x4x2x1]
- transform SyN[0.1,<r>,0]
- metric CC[<referenceImage>,<subjectImage>, 1, 4]
- smoothing-sigmas 8x4x2x1x0vox --shrink-factors 16x8x4x2x1 --convergence [500x500x500x500x100, 1e-6,10]

The value of the parameter <r > was increased stepwise from 3 up to 28.

#### Local (Non-Linear) Registration with *elastix*

We employed the B-Spline transformation model along with the adaptive stochastic gradient descent optimizer along with random image intensity sampling (Klein et al. 2009b; Klein and Staring 2015) and the NCC similarity metric of the *elastix* toolbox (Version 4.7). The most important parameter was the size of the B-Spline grid spacing, which implies a B-Spline regularization on the deformation field. *elastix* provides the option to (randomly) sample image intensities in a sub-region of the image, implementing a local similarity metric comparable to the ANTs SyN local similarity metric. A local similarity metric can be superior to a global in case of intensity inhomogeneity (Avants et al. 2008; Klein et al. 2008), e.g. caused by imaging artifacts/image inconsistencies (inconsistent intensity normalization of tilewise image acquisition or inconsistent staining of samples).

*elastix* registration with sampling from a random sub-volume for each iteration failed for this type of image data. We suppose that this is due to the inconsistent yet abundant presence of structures, such as nerve strings, outside of the brain, and hence did not use random sampling.

For *elastix*, the best image registration accuracy was achieved with a B-Spline grid spacing of 12 μm and random intensity sampling on the entire image domain. In contrast to ANTs, the employed stochastic gradient descent optimizer does not have a convergence based stopping criterion. Therefore we adapted the number of iterations of the *elastix* optimizer The computation of one pair-wise registration took between 3 and 4 min.

### An Initial Experiment Motivates the Use of *elastix*

We conducted an initial registration experiment to compare the performance of two registration algorithms of different underlying methodology, ANTs and *elastix*. The landmark registration error (LRE) of ANTs decreased with increasing value of the regularization parameter r. For ANTs, we obtained the best mean LRE of 3.33 μm ± 0.57 with parameter *r* = 28, which is slightly better than the 3.54 μm ± 0.54 LRE achieved by *elastix*. However, the corresponding maximum LRE error was 10.26 μm ± 7.76 for ANTs and 8.29 μm ± 1.2 for *elastix*.

On the evaluation data, we obtained comparable results with both registration methods: They exhibited a difference in mean LRE of only about 0.2 μm (average voxel size: 0.5 × 0.5 × 2.0 μm). However, the registration with ANTs was computationally much more expensive (> = 8 h vs. 4 min). Our aim is to provide an accurate and fast image registration approach for batch processing of existing large scale image data (on computing clusters) as well as a fast registration of new brain samples to a standard brain template (on personal computers). Based on our results of the comparison of the state-of-the-art registration methods for biomedical images, we opted to employ the *elastix* image registration toolbox (CPU implementation for cluster computing, GPU implementation for small data sets) for the generation of a standard brain template and the registration of subject scans to the template.

### Template Generation Methods

There are several approaches reported in the literature for generating a representative brain template that serves as a reference for all individual brains. The simplest solution is to select an image of an individual brain sample. Other approaches aim to calculate an average image template. The motivation for using an average-morphology template is based on geometrical aspects regarding the required deformable registration of many sample brain images to the template image. An average-morphology template is intended to represent the expected mean morphology of a population, and therefore it should require less deformation to map any sample image of the population to the average-morphology template than to any randomly selected individual brain image template.

Rohlfing et al. evaluated the impact of atlas selection strategies and found that an average-morphology template can indeed yield better registration accuracy (Rohlfing et al. 2004). The average-morphology template method (average “brain shape”) evaluated was proposed by Ashburner (2000) and is based on an iterative approach. In the first iteration, one arbitrary individual image is selected as reference image and each of the remaining images is registered to the reference image using affine transformations. Based on these transformations, an average intensity image is calculated. In the second iteration, all images are registered to the average image using nonrigid transformations. A new average intensity image is calculated based on the nonrigid transformations and used as the new reference image. This procedure is repeated until convergence. The authors state, that the shape of the arbitrary reference image does not predetermine the shape of the resulting final average image because the first iteration of the algorithm is an affine registration only.

Peng proposed an approach for generating an optimized ‘average’ template for the adult *Drosophila* brain (Peng et al. 2011). The employed registration method is landmark-based and inherently uses a quality measure to verify the overall landmark matching. The first step of the template generation is the selection of one image of a real brain as a target brain image, T_R_. Next, a large set of images is aligned globally to T_R_ with an affine registration. Those images that are aligned with a quality score larger than 75% are chosen for the generation of an average template brain. These automatically selected 256 images are then deformably registered to T_R_ and a new average template target brain, T_A, is generated by computing the mean image of the 256 deformably registered images. In this manner, local intensity inhomogeneities of the initial real image are rectified. Similar to Rohlfing et al. (2004), Peng et al. (2011) showed that the registration accuracy with the generated template is significantly improved compared to the registration with an individual brain image.

Van Hecke et al. (2008) compared a *subject based (SB)* with a *population based (PB)* template construction framework for inter-subject diffusion tensor magnetic images of the human brain. Both approaches explicitly construct an average shape space by averaging spatial transformations obtained by deformable image registration. In the following, the images of the different subjects are denoted as I_i_ (with *i* = 1, …, N_S_, and N_S_ the number of subjects). The deformation field that warps image I_j_ to image I_i_, is then defined as T_ij_.

The *SB method* is based on the calculation of the nonlinear transformations T_ij_ of all data sets I_j_ to a specific data set I_i_ of the subject group, which was selected as the initial reference image. Thereafter, the mean deformation field of the initial reference image I_i_ to all other data sets I_j_ of the group is computed, that is, the transformation of the initial subject space to the average space of the population. Next, all images I_j_ of the subject group are transformed to the final template space by the composed transformation T_ij_ (T_i_). Finally, the warped images Ĩ_j_ are averaged to compose a SB template in the average space of the population.

In the *PB method*, nonlinear transformations T_ij_ are calculated between all images I_i_ and I_j_ (with j = 1, …, N_S_). Subsequently, all N_S_ images I_i_ are transformed to the average space of the population with a specific mean deformation field T_i_ that is calculated as the average deformation of this data set I_i_ to all other images. Finally, the warped images Ĩ_j_ are averaged to compose the PB template.

### Generation of the Larval Standard Brain Template for *Drosophila*

We chose the PB approach (van Hecke et al. 2008, van van Pelt 2013) to generate a template image of the larval brain (see discussion for a motivation of our choice).

#### Image Data for Template Generation

From a large data set (Li et al. 2014), an expert manually selected 20 image stacks of good staining quality (DataSetGoodQual) that should 1) clearly show all important structures in each channel (NP, NT, GE), and 2) be morphologically representative for the population of the large data set.

#### Registration Framework for Template Generation

For the pairwise registrations in the PB template generation, we optimized an image registration framework consisting of the following four stages.

Two images are involved in each registration process. One image, the moving image, is deformed to fit the other image, the fixed image. The choice of the registration components, such as the similarity metric, is equivalent to the experimental setup for comparing registration algorithms.

1. *Nonlinear histogram matching.* For each registration, the intensity distribution of the moving image is nonlinearly matched to the intensity distribution of the fixed image. We employed the method described in (Nyul et al. 2000).
2. *Z-flip transformation.* Samples of the larval brain can be placed on the object plate in anterior or posterior position, i.e. the images acquired might be flipped in the Z-axis with respect to each other. *elastix* affine registration was not able to recover such large flip/rotation transformation and got stuck in a local minimum in the initial brain positioning. We therefore conducted two separate affine registrations where we initialized one with a transformation that flipped the image in Z-axis direction.
3. *Affine registration*. Of both conducted affine registrations, the transformation was selected that achieved a superior matching according to the similarity measure after the final iteration.
4. *Deformable registration.* The deformable image registration process is initialized with the affine transformation selected in the previous stage.

#### Generation of the Template

After computing the pairwise registrations for all 20 images, we constructed the template average space with the PB method (van Hecke et al. 2008, see Introduction). All images were transformed to the average space and then fused into the final template image by computing the voxelwise arithmetic *mean* of the intensities. We propose a modification of the “classical” image fusion step: To enhance image quality, compute the voxelwise *median* instead of the arithmetic mean. In the Results section, we compare both variants.

In order to generate a template for the tracts morphology, we applied the image transformation obtained by registration of the neuropil channel to the tracts channel.

### Subject-to-Template Registration

Subject-to-template registration refers to the registration of an imaged brain of a subject of a population to the representative template of that population. This registration process is required to construct a standard model of the larval brain, i.e. a common spatial reference framework for analysis and data mining.

#### Registration Framework

In order to obtain optimal image registration performance, the registration framework needs to be adapted to the particular registration scenario. We extended and adapted the registration framework employed for the template generation to the subject-to-template registration scenario.

#### Global Registration

The global registration was extended by one additional registration stage to cope with large rotations and the previously affine transformation was replaced by a similarity transformation. The difference between an affine and a similarity transform is that the latter only allows uniform scaling whereas the affine transform allows non-uniform scaling and shearing. Both are linear yet non-rigid transformations. DataSetGoodQual, which we used for the generation of the brain template, contains scans that have little rotation in the X-Y imaging plane, however, the other data sets contain scans with larger rotations which could not be recovered by an affine intensity based registration. To cope with large rotation, we added a registration stage that aligns images based on the Signed Distance Transform (SDT). Further, there were cases in the DataSetRandomQual where the affine registration produced a wrongly stretched image that resulted in insufficient initialization and failure of the deformable registration process.

The framework for global registration consists of the following six stages:

1. Dorsal-ventral alignment by a translation transformation to the geometrical center of the template image and flipping of the dorsal-ventral direction if the center of mass is located on the bottom half of the image.
2. *Z-flip transformation.* Equal to template generation approach.
3. *SDT feature-based registration* to cope with possible large rotations in the X-Y imaging plane. First, the image intensity is rescaled such that image acquisition block artifacts in the background are excluded. Then a coarse image mask of the brain is computed by morphological opening operations on a half-resolution resized and binarized image in order to exclude or minimize the size of artifacts in the background and at the border of the brain to the background. Next, the signed distance transform is computed on the computed binary image mask to obtain a more distinctive image pattern for rotation alignment. In a similar manner, a SDT image is computed for the brain template image. However, the background of the median fused template is homogeneous, and hence a precise image mask can be obtained by simple intensity thresholding. Finally, both SDT images are used as input images for intensity-based registration along with the MMI metric and the similarity transform.
4. If the STD-based registration fails (similarity measure <0.4), we also perform an intensity-based registration. We select the intensity-based registration if it is successful (similarity measure ≥0.4). Otherwise, we select the method that achieved the higher similarity measure and issue a registration failure warning to indicate that no method passed the 0.4 threshold.
5. *Neuropil image registration* with similarity transform and NCC image metric.
6. *Composition of the final global transformation* based on steps 1)-5). The final global transformation is used to initialize the deformable registration.

#### Deformable Registration

In subject-to-template registration, the fixed image is always the template image. The generated median fused template image provided the option to compute a precise image mask of the brain. We therefore used an image mask in the deformable registration. An image mask allows us to compute more robust measurements of image similarity because possibly confounding image information in the background can be excluded, i.e., the similarity measurement is most accurate because it is focused on intensity information in the region of interest. Moreover, with the help of an image mask it was possible to reliably employ a local similarity metric which previously failed when applied on the entire image domain.

For the deformable image registration, we employed a local NCC similarity metric. An isotropic sub-region (cubic sub-volume of 20 μm edge length) was chosen for intensity sampling.

### Automatic Quality Assessment and Semi-Automatic Registration Adjustment

#### Automatic Registration Quality Assessment

While the correlation between template and registered subject captures the global registration error, such a global measure often neglects local errors. We observed two types of local registration errors for images with weak staining, one at the terminal part of the ventral nerve cord (VNC) and one in the thoracic nerve region (s.a. Results). Similar to Muenzing et al. (2012), we developed statistical image descriptors for specific regions to detect and quantify local misalignments.

The descriptors of the VNC terminal (VI) and the thoracic nerve (TI) regions are confidence values that assess the similarity of the respective regions in the template and in the registered image stack. Based on a training data set, we determined that confidence values below 50% were indicative of a major registration error in the respective region. Hence, a confidence value below 50% for VI or TI initiated a manual error correction procedure.

##### VNC Terminal Error Indicator (VI)

The mutual information score (as defined in Mattes et al. 2003) was calculated between the registered subject image and the template image on spherical regions with a radius of 10 μm at two terminal VNC landmark positions defined in the template image. The mutual information score was expressed in percent of the maximum observed score (for all scans), such that the VNC terminal that was registered best resulted in a VI value of 100%.

##### Thoracic Nerve Error Indicator (TI)

The magnitude of the Eulerian strain tensor (Mase 1970) was calculated on the deformation field at spheres with radius of 35 μm at all six thoracic nerves defined in the template image. The strain magnitudes were then normalized by subtracting the median strain magnitude (of all scans) and dividing by the median absolute deviation. The normalized strain, denoted by S, was scaled to the range from 0 to 100%. We found that the 95% percentile of the normalized strain measurement was the most reliable indicator of local image distortion caused by failed registration of the thoracic nerve region.

In addition, the mutual information score, M, was computed as for the VI case: Here, at all six thoracic nerves a region with a radius of 15 μm was extracted. Both M and S range from 0 to 100%. We combined them to compute the thoracic nerve indicator TI as M*S/100.

#### Semi-Automatic Registration Adjustment

If a registration achieves a confidence value below 50% for TI or VI, landmarks are set manually in the subject image (using the landmarks plugin in Fiji: Schindelin et al. 2012). In case of the described VNC registration failures (VI), two landmarks, i.e. the positions of the V6 nerve entries are sufficient to guide the semi-automatic registration. Reliable correction of misalignments at the thoracic nerve region requires the annotation of all six thoracic nerve entry points. In case of registration failure of both VNC and thoracic region, eight landmarks need to be annotated manually. Corresponding landmark annotations for the template image need to be provided only once.

The framework of semi-automatic deformable registration is based on an approach where two similarity metrics drive the registration process (*elastix* multi-metric registration: Klein and Staring 2015, Chapter 6). In addition to the previously employed NCC metric, the Euclidean distance of the corresponding points (Baiker et al. 2011) is computed between the landmarks in the template and the manually defined landmarks in the subject image. Both metrics are computed simultaneously in a multi-resolution setting, and proper weights have to be found to balance these two metrics in order to avoid image distortions, especially in the scenario where few landmarks define the correspondences of only a small part of the region of interest. We achieved robust registration results by using relative weights, that is, instead of balancing the similarity metric measurements, the gradient of the similarity metric is balanced to ensure that the registration process is smoothly guided by both landmark correspondence and structural intensity based image information.

### Image Data

We used image stacks of the Janelia database of the larval Drosophila CNS (wandering third instar; data by courtesy of co-author JWT). Data recording followed the protocol described in Li et al. (2014): The employed labels were “mouse antineuroglian (1:50 BP-104; Developmental Studies Hybridoma Bank), rat anti-N-cadherin (1:50 DN-Ex #8; Developmental Studies Hybridoma Bank) and rabbit anti-GFP immunoglobulin (Ig) G (1:1,000; Invitrogen A11122)”. The size of the 8 bit image stacks is about 1000 × 1400 × 100 voxels (in-plane resolution: 0.5 μm, slice thickness: 2 μm, z-step: 2 μm).

## Results

### Standard Brain Template

We computed the NP template as described (Methods) and then applied the obtained transformations for NP to the NT channel to generate the NT template.

**Fig. 2:**
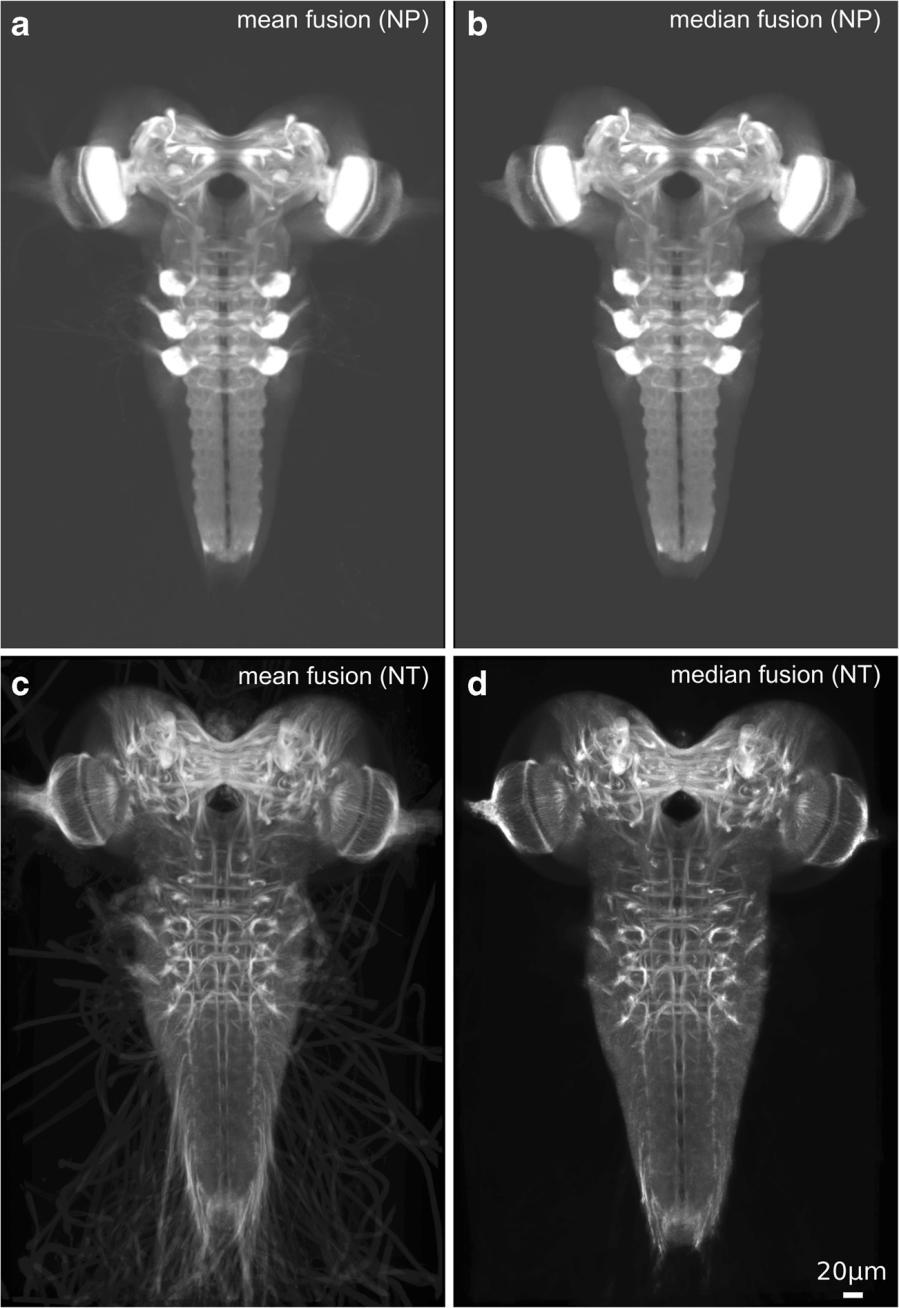
Generated template images for the NP (**a**,**b**) and NT (**c**,**d**) channels. **a** NP, mean fusion (**b**) NP, median fusion (**c**) NT, mean fusion, **d** NT, median fusion

Figure 2 shows both the NP and NT template images computed by mean intensity fusion and the median intensity fusion proposed in this work (Methods).

All templates exhibit a high level of detail. Also the NT templates (Fig. 2c, d), that were computed indirectly through warping, contain all the expected tracts that are visible in single sample brain images. Thus, the image registration framework has generated an accurate mapping of the intensity information on the neuropil channel along with robust and undistorted deformation fields. The difference between mean fusion and median fusion is most apparent in the background where diffuse blur is visible from nerve string structures in the mean fusion image, whereas the background of the median fusion template appears homogeneous.

### Subject-to-Template Registration

#### Image Data

**Fig. 3:**
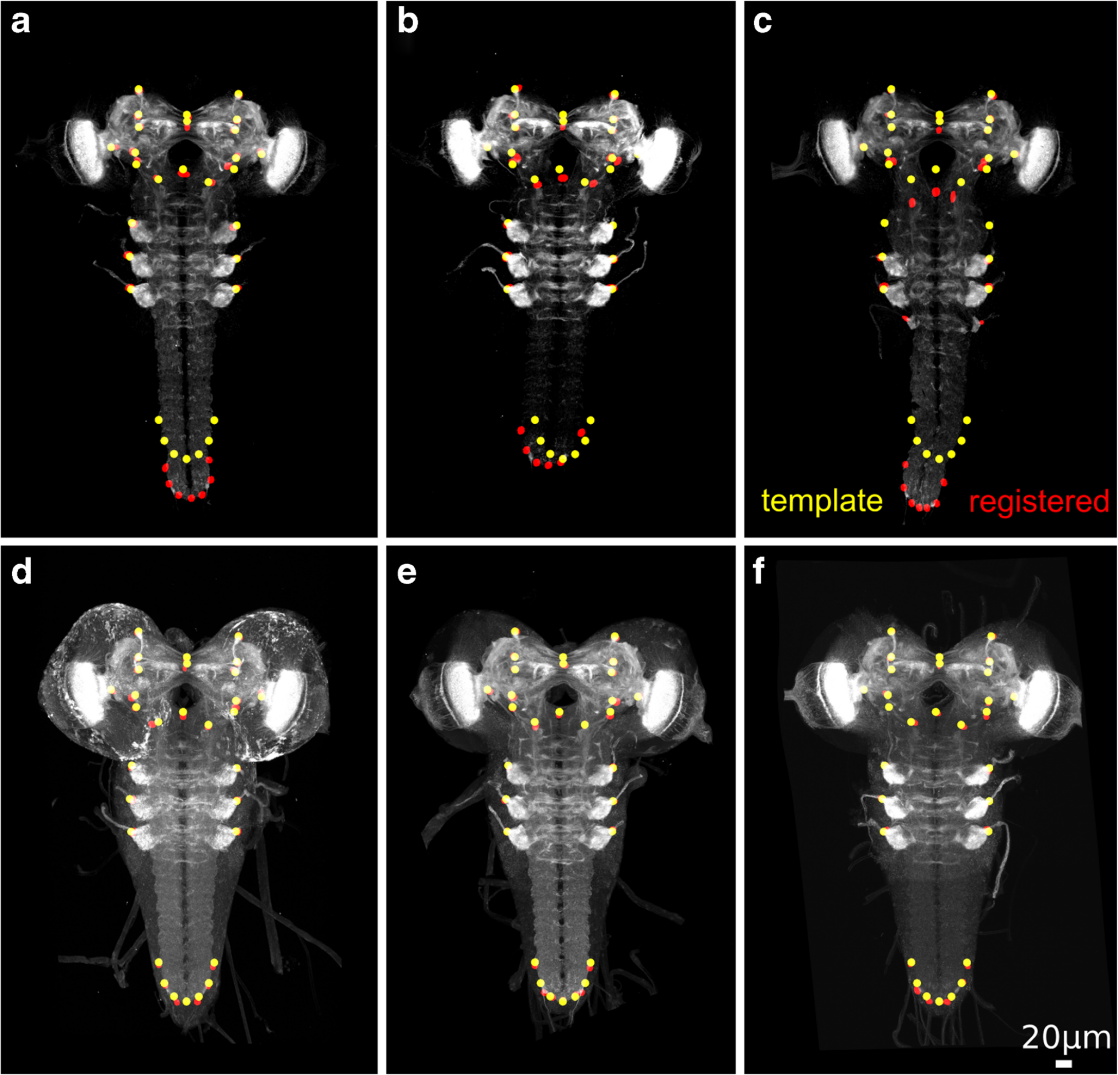
Example registrations (NP channel) from DataSetRandomQual. Red circles denote landmark positions on the registered subject image. Yellow circles refer to the corresponding landmark positions on the template. The circles have a diameter of ca. 10 μm. **a-c** poor image quality causes large registration errors. **a, b** Two cases of registration failure at the VNC terminal. **c** registration failures at both the thoracic nerve region and at the VNC terminal. **d-f** Successful registrations from DataSetMediumQual

We evaluated the subject-to-template registration on image stacks of the Janelia database of the larval Drosophila CNS (Li et al. 2014). From the database, we selected three subsets of different image quality, where DataSetGoodQual and DataSetMediumQual contain higher-quality image stacks selected by visual inspection, while DataSetRandomQual contains a random selection of stacks (Table 2, Fig. 3). A neurobiologist assessed image quality based on whether there was a low/high staining background or whether the staining was complete/incomplete. For quantitative evaluation, we established a gold standard (Methods) for the generated neuropil template and for all stacks from the three data sets.

**Table 2:** Data sets for evaluation (see Fig. 3 for example images)

Name	# images	Image quality
DataSetGoodQual	20	good
DataSetMediumQual	30	medium
DataSetRandomQual	25	randomly selected images

#### Results for Subject-to-Template Registration

Table 3 summarises the landmark registration errors (LRE) for all three data sets. The lowest LREs were obtained on the data sets with the best image quality, DataSetGoodQual and DataSetMediumQual.

**Table 3:** Evaluation of subject-to-template registration. Landmark registration errors (LRE) were averaged per registration and then the median error, the mean error and the corresponding standard deviation were calculated. The maximum error denotes the mean over the largest registration errors for each registration

Name	LRE [μm]		
	Mean ± Sd	Median	Max
DataSetGoodQual	2.5 ± 1.5	2.2	7.1
DataSetMediumQual	4.1 ± 3.6	2.9	15.2
DataSetRandomQual	6.9 ± 6.4	4.6	24.5

DataSetRandomQual contains several images with overall or partially weak staining. Here, we frequently observed two types of registration errors that resulted in partial or global distortions of the registered image (VNC error: Fig. 3a, b, thorax and VNC error: Fig. 3c). For comparison, Fig. 3d-f show successful registration results on images of medium quality.

#### Computation Time for Subject-to-Template Registration

Both, the fully automatic and the semi-automatic registration have only moderate computational requirements of 7 or 8 min, respectively, per image stack (Table 4). Computation times were measured for an exemplary set of 7 image stacks on a machine with the following configuration: Intel(R) Core(TM) i7-6700 K CPU @ 4.00GHz, 64GB RAM, NVIDIA GeForce GTX 980 Ti, Windows 7 64 bit operating system.

**Table 4:** Average computation times for subject-to-template registration (entire image registration pipeline including all steps) measured for CPU and GPU computation. For the semi-automatic case, computation times do not include manual interventions

Average computation times [sec]	Fully automatic		Semi-automatic	
	CPU	GPU	CPU	GPU
Linear registration	112	109	140	130
Nonlinear registration	172	145	176	173
Combining transformations	42	42	42	42
Warping of image channels	79	78	76	75
Automatic quality assessment	35	35	36	36
Sum [sec]	440	409	470	456
Sum [min]	7	7	8	8

### Semi-Automatic Registration Correction

We evaluated the semi-automatic registration (Methods) on DataSetRandomQual. There were three cases of the described registration failures: two VNC and one thoracic case. Table 5 lists the registration error of the subject-to-template registration for each image after fully automatic and after semi-automatic registration of the three failed registrations. The average error of the partially failed registrations (bold font) was reduced from 12.9 μm to 5 μm and the overall registration error of the data set was reduced from 6.4 μm to 5.4 μm. Only few landmark annotations (2 for VNC errors, 6 for thorax errors) were required to correct a partially failed registration.

**Table 5:** Registration errors on DataSetRandomQual with fully automatic and semi -automatic registration

Scan	LRE [μm]							
	Automatic				Semi-automatic			
#	Mean	Sd	Median	Max	Mean	Sd	Median	Max
1	7.9	(7.5)	5.4	29	7.9	(7.5)	5.4	29
2	4.5	(4.0)	3.2	15	4.5	(4.0)	3.2	15
3	6.9	(5.8)	4.6	19	6.9	(5.8)	4.6	19
4	6.6	(6.1)	4.0	21	6.6	(6.1)	4.0	21
5	7.9	(6.3)	5.6	31	7.9	(6.3)	5.6	31
6	7.9	(8.2)	3.7	32	7.9	(8.2)	3.7	32
7	4.8	(3.9)	3.7	15	4.8	(3.9)	3.7	15
8	4.2	(3.7)	2.5	15	4.2	(3.7)	2.5	15
9	5.9	(5.9)	3.5	23	5.9	(5.9)	3.5	23
10	5.2	(4.8)	3.4	21	5.2	(4.8)	3.4	21
11	4.4	(3.5)	3.5	18	4.4	(3.5)	3.5	18
12	**13.3**	**(18.2)**	**5.2**	**69**	**7.0**	**(5.0)**	**3.3**	**15**
13	5.4	(2.8)	4.1	14	5.4	(2.8)	4.1	14
14	4.7	(5.2)	2.4	24	4.7	(5.2)	2.4	24
15	**14.0**	**(19.2)**	**3.9**	**55**	**3.9**	**(2.1)**	**3.4**	**9**
16	4.1	(3.5)	3.0	16	4.1	(3.5)	3.0	16
17	4.2	(3.0)	3.3	12	4.2	(3.0)	3.3	12
18	3.9	(2.8)	3.2	12	3.9	(2.8)	3.2	12
19	**11.4**	**(14.8)**	**4.5**	**49**	**4.2**	**(2.9)**	**3.9**	**15**
20	4.6	(3.1)	3.5	14	4.6	(3.1)	3.5	14
21	5.5	(6.4)	3.3	27	5.5	(6.4)	3.3	27
22	6.5	(5.4)	4.8	21	6.5	(5.4)	4.8	21
23	3.8	(2.9)	2.7	13	3.8	(2.9)	2.7	13
24	6.7	(7.1)	4.4	27	6.7	(7.1)	4.4	27
25	4.5	(2.8)	3.7	13	4.5	(2.8)	3.7	13
Average	6.4	(6.3)	3.8	24.2	5.4	(4.6)	3.1	18.8

Partially failed registrations: bold font

### Complete Subject-to-Template Registration Workflow (Including Error Correction)

In order to evaluate the frequency of cases where semi-automated registration correction is needed and to assess the reliability of the automatic registration error detection and semi-automatic registration adjustment, we performed an evaluation of the entire registration workflow, i.e. the subject-to-template registration and automatic registration error detection followed by semi-automatic registration correction (Methods), on a large data set.

We registered the first 250 scans (ordered by scan ID) of the public data set (Li et al. 2014) to the previously generated neuropil template. All 250 registrations underwent a quality check by merging the registered NP channel with the NP template in different colors (green and magenta) to then visually inspect deviations by experts in neurobiology (Methods). Registrations that partially failed were corrected using the semi-automatic registration framework (Methods).

222 out of the 250 automatic registrations passed the quality check by neurobiologists. The remaining 28 scans, about 11% of all scans, were corrected using the semi-automatic error correction method.

**Fig. 4:**
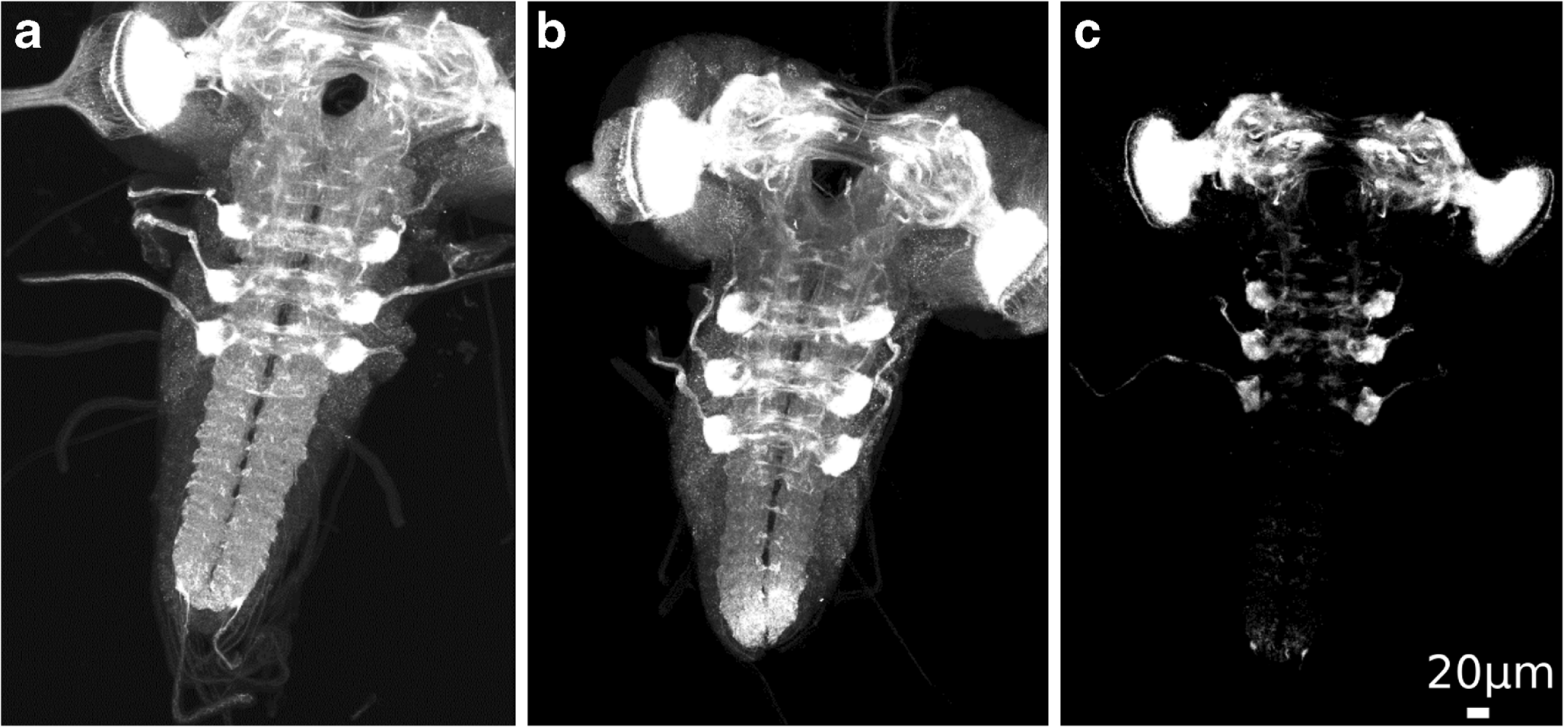
Failed registrations of images (NP channel) using the semi-automatic registration framework. 8 images of the entire set of 250 images did not allow for a successful registration, either because images were incomplete (**a**), had a high staining background (**b**), or because of missing staining throughout the ventral nerve cord (**c**)

After semi-automatic error correction, 20 of the 28 failed registrations passed the human-observer quality check, i.e. overall 242 of the total of 250 scans could be registered successfully. The remaining 8 scans, for which the registration failed, can be attributed to problems with data acquisition: They either suffered from bad image quality or did not cover the entire brain. Figure 4 shows three examples for failed registrations.

#### Automatic Vs. Human Expert Quality Check

We also compared the automatic quality assessment based on the VI and TI criteria (Methods) with the quality check by a human expert. The confusion matrix in Table 6 shows that all but one of the 28 human-rejected registrations were also predicted as “rejected” by the automatic quality assessment, while seven registrations were falsely predicted as “rejected”.

**Table 6:** Confusion matrix for the comparison of human quality check (accepted/rejected) and automatic quality check based on the VI and TI criteria (predicted as accepted/rejected)

	Predicted as accepted	Predicted as rejected	sum
Accepted	215	7	222
Rejected	1	27	28
Sum	216	34	250

### Biological Use Cases: Overlap of Registered Expression Patterns

To assess the quality of the proposed registration framework, we addressed two different biological use cases. A key application will be the visualization and identification of overlapping expression patterns in specific neuropil regions or even on the single cell level.

**Fig. 5:**
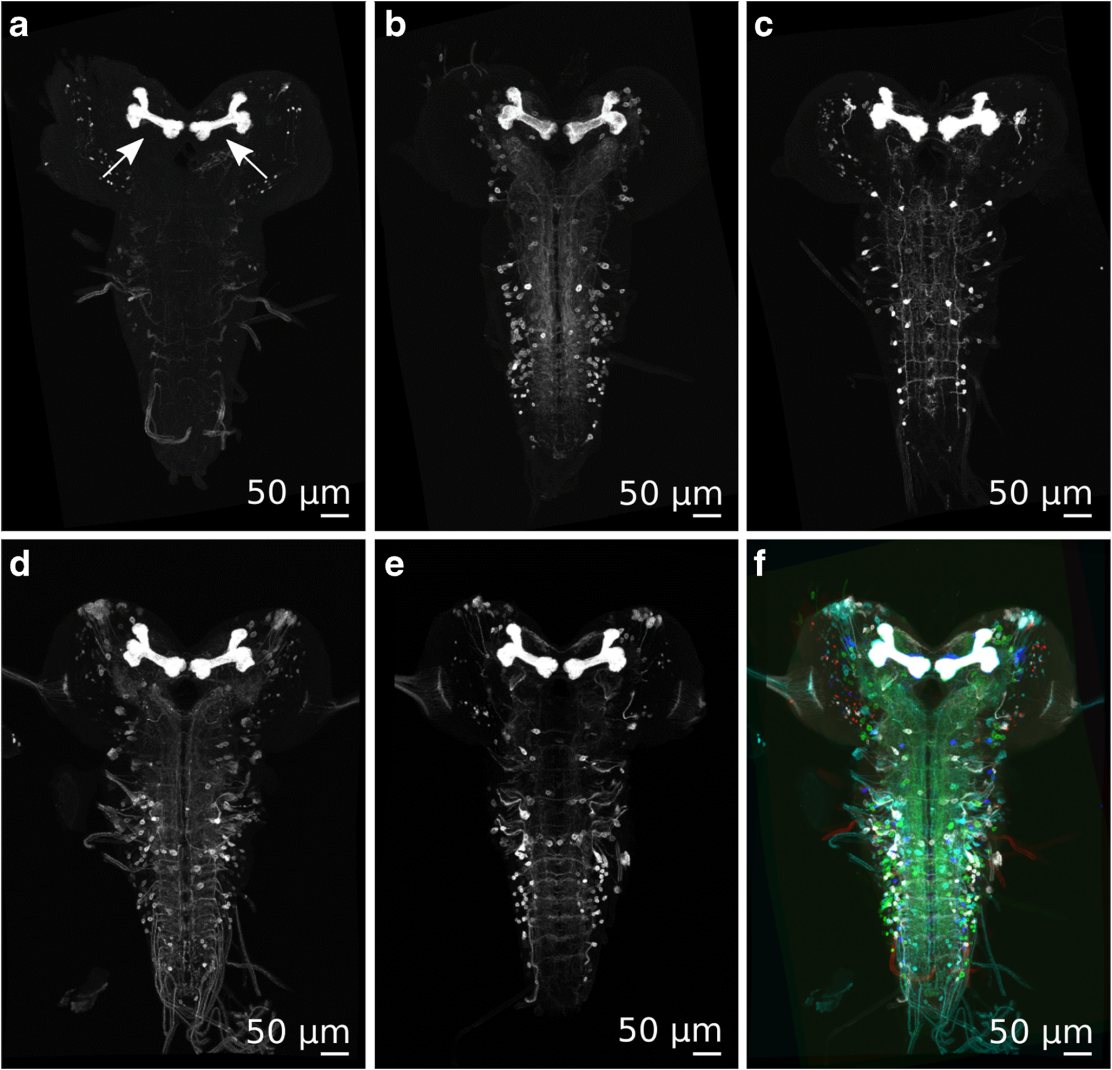
**a-f** Five different registered GE images that all label the mushroom bodies (marked by arrows in **a**). **f** All five registered images merged into one (data: Pauls et al. 2010)

We chose the larval mushroom body, a central brain region involved in learning and memory, for a closer inspection on the neuropil level. Figure 5 shows five different expression patterns (Fig. 5a-e) of different Gal4 lines that all label the larval mushroom body (Pauls et al. 2010). When all registered expression patterns are merged into one image, the mushroom body (marked by arrows) appears as a precise overlay (Fig. 5f), demonstrating the accuracy of the registration procedure.

**Fig. 6:**
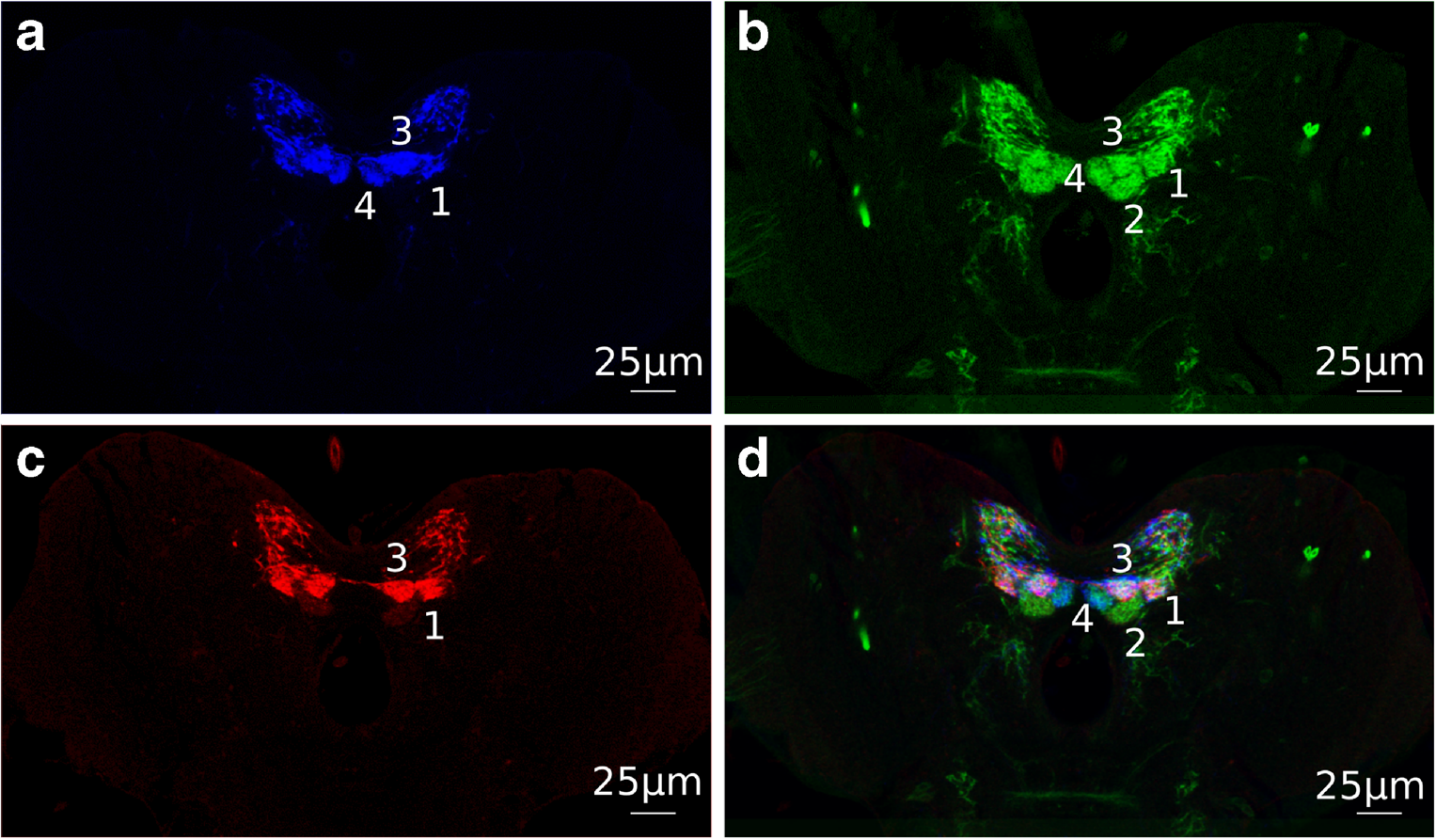
Registered Gal4 lines images. **a** 58E02 (blue), **b** 64H06 (green), **c** 30G08 (red) **d** All three registered Gal4 line images merged. All pictures show a zoom-in on both brain hemispheres. The three different GAL4 lines were reported to label different sets of four central dopaminergic neurons. The presynaptic areas of the four neurons are marked by numbers (data: Rohwedder et al. 2016)

Figure 6 focuses on four individual dopaminergic neurons that were reported to be all (expression pattern of 64H06) or partially (58E02 and 30G08) included in GAL4 lines (Rohwedder et al. 2016). After registration, the four different innervations of each neuron are clearly visible. The four different neurons precisely adjoin each other, appearing as four distinct ball-like structures reflecting the presynaptic areas of each neuron (Fig. 6d).

## Discussion

### Template Generation

In principle, a single brain scan of high quality could serve as the template against which all other brain scans are registered. However, using a population mean instead of a single brain has been reported to yield better registration results (Rohlfing et al. 2004; Peng et al. 2011), and, indeed, a population mean template is the minimizer of the average deformation magnitude needed for registration to the template.

In contrast to adult brain images (see e.g. Peng et al. 2011), the acquired images of the larval brain show large deformations introduced by sample preparation or positioning of the sample on the object plate. It is therefore crucial to use accurate nonlinear mappings for all aspects of the template generation process in order to obtain a good representative template with respect to morphology and image quality (sharpness and conservation of details).

The SB and PB method (van Hecke et al. 2008) appears to be best suited for our data since both SB and PB generate a template of average shape, as well as an average intensity appearance. Van Hecke et al. (2008) stated that there is a potential bias in the SB approach towards the selected single reference image, and they demonstrated on synthetic data that the PB approach is able to outperform the SB method by about 30% on diffusion tensor images.

We hence opted for the PB approach to generate a template image of the larval brain. Deviating from the original PB approach, we used the median instead of the mean in the final image fusion step (Methods), which led to visible improvements of the template image (Fig. 2).

### Choice of Registration Algorithm

The initial evaluation (Methods) of the two most promising state-of-the-art registration methods, ANTs and *elastix*, showed that LRE performance on gold standard data was similar for both methods. However, computational costs were in the order of several hours for ANTs, while *elastix* only required minutes for the same task. The high computational demands of ANTs would be in conflict with our goal of providing an accurate and fast image registration approach that allows users to align their own expression patterns to the template brain using standard computing hardware. As the data sets in this domain can contain hundreds or thousands of scans, we decided to employ *elastix* as the registration algorithm for *larvalign*.

### Quality of the Registration Results

We have provided extensive empirical evaluation of our registration framework (Results). For measuring registration accuracy, we relied on the landmark registration error (LRE) that is based on a gold standard with landmarks annotated by an expert (Methods).

We could thus obtain a reliable measure of accuracy, limited only by biological and intra-rater variance, for three data sets of manually annotated brains.

The proposed registration achieved a LRE of 2.5 μm ± 1.5 μm (Table 3) for the data set with the highest image quality (DataSetGoodQual). This is in the order of the human intra-rater variation (2.0 μm ± 1.2 μm) that sets a lower limit for the landmark-based error that can be measured.

For comparison, the size of a complete third instar larval CNS is about 500 × 200 × 200 μm^3^ (Lemon et al. 2015), the first order center of the olfactory pathway has a diameter of about 28 μm and the subesophageal zone, the first order integration center of the gustatory pathway, has a width of about 116 μm.

The LRE was 6.9 μm ± 6.4 μm (Table 3) for the worst image quality within the representative data set (DataSetRandomQual). It should be taken into account that the intra-rater variance was measured on high-quality data, i.e. the intra-rater variance as a baseline error could also be higher for DataSetRandomQual. However, the high standard deviation for DataSetRandomQual is an indicator for a number of failed registrations (see Fig. 3).

Even with the most sophisticated registration algorithms, registration failures can occur if image quality is insufficient. We have thus introduced a semi-automatic approach to detect and correct failed registrations (Methods) that could reduce the LRE for DataSetRandomQual to 5.4 μm ± 4.6 μm (Table 5).

For further evaluation, we relied on an expert vote for each of 250 brain registrations from a larger data set. We employed this larger data set to evaluate the entire registration framework, i.e. registration followed by semi-automatic error correction, in a realistic high-throughput application scenario. According to the expert vote, only 8 out of 250 registrations were not acceptable, and there were obvious reasons, such as incompletely scanned brains, for the registration failures (Fig. 4).

Overall, the proposed registration framework is thus suitable for its intended application purpose, and robust in the face of variable image quality.

### Biological Applications

In addition to the quantitative evaluation, application examples for mapping gene expression patterns serve as a qualitative evaluation of our registration framework. In both biological use cases (Figs. 5 and 6), precise overlay within the expected brain parts and cells was visible for the registered expression patterns, demonstrating that the achieved registration accuracy is high enough for addressing biological questions of brain organization and function in Drosophila larvae.

With the proposed registration framework at hand, it is now possible to 1) label every neuron in a larval meta-brain where different neurons have been labelled in different GAL4 lines, 2) quantify how conserved expression patterns are between individual larvae, 3) estimate neural connectivity through detection of neuron overlap.

Going beyond the alignment of gene expression channels shown here, the same registration framework could also be used to align whole-brain brain activity snapshots as enabled by the CaMPARI calcium indicator (Fosque et al. 2015).

## Conclusion

We have introduced and evaluated computational methods to 1) generate a reference template of the larval brain of *Drosophila*, and to 2) register individual brains onto the template, enabling the mapping of expression patterns from different larval brains into the same reference space.

Our methods allow for a high degree of automation, but also include a semi-automatic correction for failed registrations. High-throughput processing of large data sets is thus augmented by manual treatment of problematic cases, facilitating the creation of a curated database of registered expression patterns with a high standard of quality.

## Information Sharing Statement

A software implementation of the registration framework, *larvalign*, is available at: https://github.com/larvalign/larvalign/releases/tag/v1.0

The *larvalign* (RRID:SCR_015815) distribution contains open source software: *elastix* (RRID:SCR_009619, Klein et al. 2010) for image registration, ITK-SNAP (RRID:SCR_002010, Yushkevich et al. 2006) for image pre- and postprocessing, and Fiji (Schindelin et al. 2012) for landmark annotation, image format conversion, and image visualization.

The image data sets are available at https://github.com/larvalign/Data and https://github.com/larvalign/larvalign/tree/master/ImageData

Requirements:

- Matlab runtime (available for free at: https://www.mathworks.com/products/compiler/mcr.html)
- Memory requirements depend on image size. For the image stacks used in this work, we recommend a minimum of 16GB RAM with at least 8 GB RAM available for the *larvalign* software. *larvalign* will work with less, however at the cost of longer computing times.
